# Outcomes of hip fracture treatment with intravenous morphine and with other analgesics: postoperative analgesic medical expense, severity of pain and hospitalisation—a retrospective study

**DOI:** 10.1186/s13018-023-04328-w

**Published:** 2023-12-06

**Authors:** Rapeepat Srichan, Phichayut Phinyo, Krittai Tanasombatkul, Puwapong Nimkingratana

**Affiliations:** 1 Department of Orthopaedics, Faculty of Medicine, Chiang Mai University, Chiang Mai, Thailand; 2 Center for Clinical Epidemiology and Clinical Statistics, Faculty of Medicine, Chiang Mai University, Chiang Mai, Thailand; 3 Department of Family Medicine, Faculty of Medicine, Chiang Mai University, Chiang Mai, Thailand

## Abstract

**Aims:**

This study compares the postoperative medical costs and outcomes of hip fracture patients treated with intravenous (IV) versus other analgesics (weak opioids, NSAIDs or acetaminophen).

**Methods:**

We performed a retrospective study at a tertiary hospital in Thailand, examining 1,531 patients who underwent hip fracture surgery between 2009 and 2020. We analyzed data on analgesic usage, costs, pain scores, and adverse effects.

**Results:**

In the study of 1531 patients, 63% of patients received as-needed analgesics, and 37% received preemptive prescriptions. In both groups, IV morphine was the predominant choice. The mean cost for the IV group was marginally higher than the other analgesics group ($2277 vs $2174). The other analgesics group had a significantly higher consumption of acetaminophen and selective NSAIDs (*p* = 0.004). Pain scores were similar across both groups, but the IV group had a significantly higher incidence of gastrointestinal side effects (24% vs 10.5%, *p* < 0.01).

**Conclusion:**

The choice of IV or other analgesics in treating hip fractures affects analgesic usage, side effects, medical costs, and patient outcomes. Further studies across different regions are recommended.

**Supplementary Information:**

The online version contains supplementary material available at 10.1186/s13018-023-04328-w.

## Introduction

Among osteoporotic fractures, hip fractures are associated with significantly high morbidity and mortality rates as well as high medical costs [1–3]. The hip fracture rate approximately doubles each decade after age 50. The incidence is also up to two to three times higher in women than in men over age 50 [4–6]. According to a study by an Asian Federation of Osteoporosis Societies (AFOS) member, the number of hip fractures in Asia is projected to reach 2.56 million by 2050, up from 1.12 million in 2018 [7]. For most hip fractures, operative treatment is recommended when possible because of the relatively high risk of death with nonoperative treatment [8]. Surgery for hip fracture should be performed within 48 h because a shorter time to surgery results in better clinical outcomes for patients [9].

A key concern after hip surgery is postoperative pain because it is associated with delays in ambulation and prolonged hospital stays, as well as poor functional outcomes and reduced quality of life [10–12]. There are many modes of pain control for patients with hip fractures, including oral and parenteral systemic analgesia, e.g., paracetamol, nonsteroidal anti-inflammatory drugs, and opioids, as well as epidural and spinal anesthesia and peripheral nerve blocks [13, 14]. Opioids are one form of multimodal pain control in hip fractures, and opioid use has been increasing substantially worldwide [15]. Reasons for the increase include that opioids are practical, effective, relatively low cost, and easy to use [16]. However, the elderly are more vulnerable to their side effects, including constipation, drowsiness, respiratory depression, nausea, and postoperative delirium [17, 18]. Balancing the advantages and disadvantages of morphine prescription may mean that using other pain control modalities or combinations of analgesics may be a preferable alternative in most cases. Several studies have reported on the effectiveness of intravenous acetaminophen in pain control protocols for hip fractures compared to the standard regimen. Protocols involving intravenous acetaminophen have been shown to result in significant improvement in pain scores, reduction in opioid use, shorter length of hospital stay and fewer missed physical therapy sessions [19].

Other non-opioid analgesics and modalities, however, come with a higher cost. Patients have been reported to typically spend about €7,500 for epidural analgesia compared to €7,273 for morphine patient-controlled analgesia (PCA) [16]. Similarly, total hospitalization costs were found to be lower with multimodal analgesia than with intravenous opioid monotherapy: US$12,540 ± $9,564 vs. $13,242 ± $35,825 (427,363 ± 325,941 THB vs. 451,287 ± 1,220,916 THB), respectively [20]. Fewer adverse events have also been reported using multimodal analgesia [19].

Intravenous morphine is among the analgesics commonly used postoperatively, especially in hip fracture surgery. Morphine apparently has a greater ability to control severe pain compared to other analgesics [21]. Current regulations in Thailand regarding the use of analgesic drugs are not clear [22]. Intravenous (IV) morphine is a high-potency drug which, in Thailand and most other countries, can only be prescribed in a hospital. It is relatively low cost, widely available, and is commonly combined with other analgesics to achieve adequate pain control. Additionally, there is no limitation on its use in individual healthcare coverage. This study found that approximately 75% of hip fracture patients in our study hospital received morphine (unpublished data) in contrast to global use of about 99% [23].

The objective of this study was to compare IV morphine and other multimodal analgesics in terms of analgesic drug expense, length of hospital stay, postoperative complications, time to rehabilitation, pain score before and after rehabilitation, delay in ambulation, analgesic-related complications, over-and under-prescription of analgesics and functional outcomes in postoperative hip fracture patients including the types and quantities of analgesics used. The results of the study will hopefully promote discussion regarding the use and effectiveness of analgesics as well as providing support for additional safety precautions related to their prescription.

## Methods

### Study design and patient selection

This retrospective cohort study reviewed daily admissions to our center of patients with an intertrochanteric or femoral fracture from 1 January 2009 through 31 January 2020. A trained research associate collected patient’s data through a review of hospital medical records. Baseline characteristics of patients were obtained from reviews of medical charts.

The inclusion criteria were age 18 years or older on admission and having undergone a primary inpatient surgical hip fracture procedure. The exclusion criteria were patients without a hip fracture or with no procedure codes for hip fracture surgery, patients with a previous hip fracture, patients with a length of hospital stay of more than 30 days, patients who used other strong opioids and other routes of morphine consumption, and patients who underwent a local nerve block during the hip operation. The patients were divided into two groups: patients who received intravenous morphine (the IV group) and patients who received the other analgesics group. Information was obtained on all analgesic drugs administered for each patient, including oral and injection medications as well as the use of transdermal patches.

### Intravenous morphine consumption

We determined the total IV morphine each patient consumed using medical records and chart reviews, both certified by a physician and a nurse. According to hospital policy, the usage of IV morphine is also monitored by requiring that any unused IV drugs be returned to the hospital and that the amounts returned be recorded. The quantity of IV morphine used was categorized into that used before surgery, post-operation, and recovery prior to rehabilitation. We also recorded the frequency of use separately to help confirm the total consumption and to calculate the average IV morphine dosage. Notes on anesthesia use and patient medical records provided patient body weight information. We calculated the per kilogram dose by dividing the total amount used by the patient's body weight. To calculate postoperative morphine use, the quantity of postoperative IV morphine used was divided by body mass since the surgery was conducted after the first rehabilitation period.

### Other analgesics used

All other analgesics used for each patient were tracked using the hospital's analgesic drug lists for four categories of analgesic drugs: acetaminophen, non-selective NSAIDs, selective NSAIDs, and weak opioids. Each category of drug was then separated into oral drugs and IV drugs. The total consumption, total cost, and cost per unit of each of the analgesic drugs was recorded. The per unit cost by weight of each drug was used to calculate the total cost of drugs used with each patient.

Three routes are available for analgesic drug administration: oral, injection, and patch form. The oral route was the standard used for pain control while injection was used in cases of 99%. As the patch was used with only a small minority of patients, data on patch delivery was recorded but was not used in the comparison of the two study groups. Analgesic drug usage and cost in the IV and other analgesics groups were also collected and compared.

### Outcome

We examined the relationship between the level of pain in the IV and other analgesics groups as well as in-hospital outcomes, including total hospital cost, analgesic drug cost, length of stay, postoperative complications, time to rehabilitation, pain score before and after rehabilitation, delay in ambulation, analgesic complications, and functional outcomes. Based on the data available in hospital records, patients' level of pain was calculated as a numeric score on a scale of 1 to 10, where NS 1 = no pain and NS 10 = very severe pain. Pain scores were determined immediately after a patient was transferred to the ward following surgery then every 4 h until entry into the the rehabilitation program. Patients with moderate (NS 5-7) to severe pain (NS > 7) were identified and their analgesic drug use information was recorded. Physicians independently reviewed each patient's complications which had been recorded at the initial visit plus the dosages of antiemetic drugs following the International Statistical Classification of Diseases and Related Health Problems (ICD10).

### Adverse effects

Potential adverse effects of analgesic drugs, identified from *PubMed* and *Scopus*, include problems involving the respiratory system, gastrointestinal system, central nervous system and genitourinary system, as well as other adverse effects such as bradycardia, rash, itching, and falls from bed. Each possible adverse effect was matched with ICD-10 and the patient's history in the medical record following the hip fracture event. All the adverse effects are collected before using therapeutic drugs such as PPIs, antiemetics, and antibiotics drugs.

### Medication cost

Analgesic drugs used for each patient, including name, type, total cost, unit price, and total consumption, were obtained from the hospital records database. The number of drugs and the total cost of the drugs were recorded. The unit price of each analgesic drug was recorded at the time of use as some prices change over time. Trends in use of individual drugs were plotted to identify changes over time.

### Statistical analysis

Patient analgesic use was divided into two groups: analgesics prescribed as needed and those prescribed preemptively using a pain score cut point of 3. Selected patient characteristics were used in the analysis for weighted adjustment, including actual age, age > 70 years, gender, BMI, body weight, height, type of fracture, type of operation, operation time, and type of anesthesia. Hospital costs, quantity, and cost of analgesic drugs were calculated and categorized by group. The heterogeneity of unweighted and weighted groups were defined by absolute standardized difference. Differences in continuous variables between the 2 groups were assessed using weight median regression; differences between categorical variables were determined using weight risk difference regression. Associations were considered statistically significant if *p* < 0.05. Statistical analysis was performed using STATA version 16 (StataCorp, College Station, TX, USA) and Microsoft Excel (Microsoft 365, Microsoft, Seattle, WA, USA).

## Results

### Patient characteristics

**Fig. 1:**
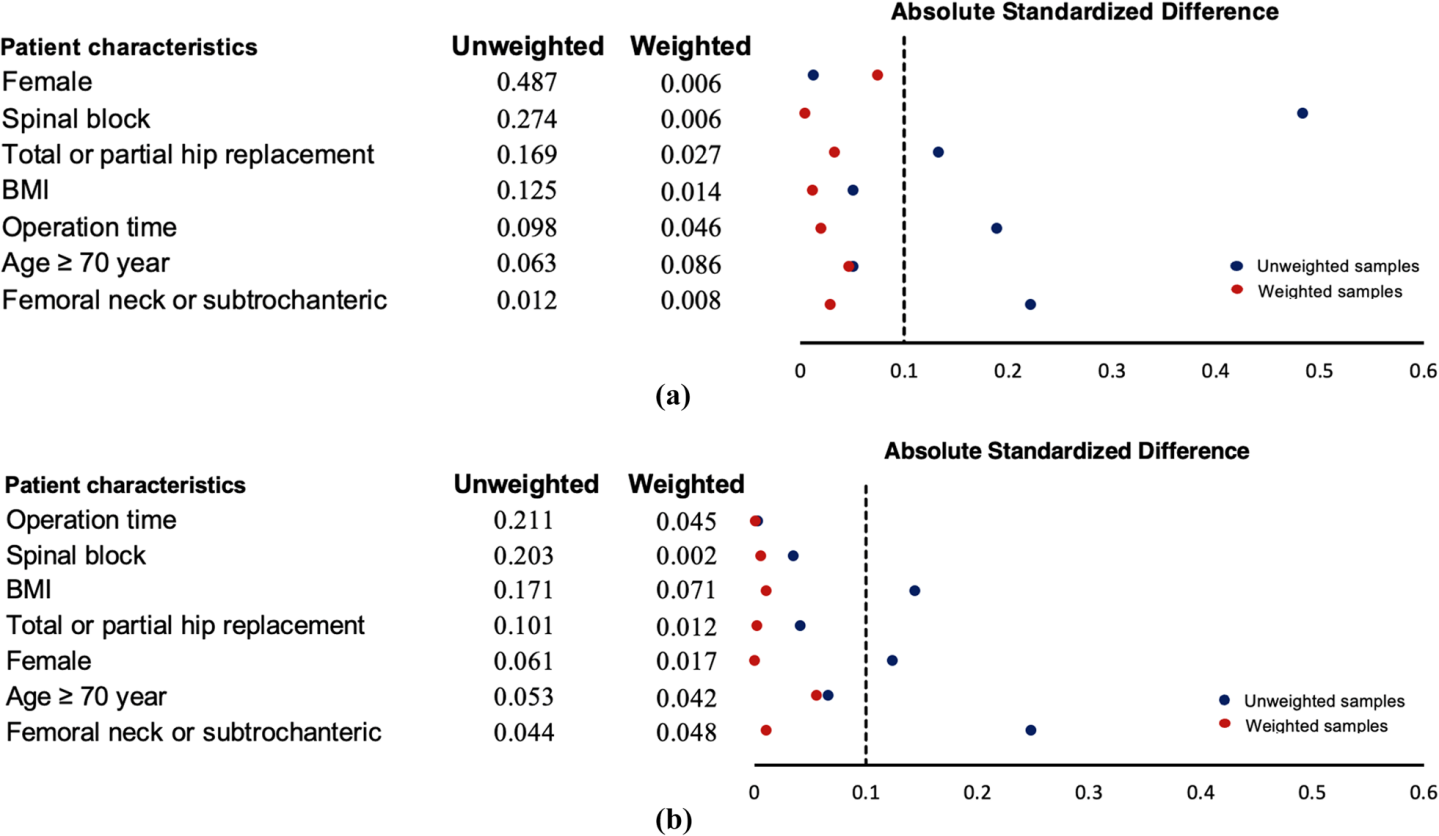
**A** Analgesic prescribed as-needed for pain control** B** Analgesic prescribed preemptively

A total of 1,531 patients age 18 years or older underwent primary hip fracture surgery in a tertiary-level medical school hospital in Thailand between 1 January 2009 and 31 January 2020. Characteristics of the patients are presented in Table 1. Of those patients, 963 were in the “prescribed as needed” analgesia group and 568 were in the “preemptively prescribed” analgesia group. Each group was further divided into an IV morphine subgroup and other analgesics subgroup. Baseline characteristics, including age, gender, BMI, body weight, height, type of fracture pattern, type of surgery, type of anesthesia, and operation time, were weighted. The absolute standardised differences were less than 0.1 in the after-operation weight group for all parameters compared to the before-operation weight (Table 1). Figure 1A,B shows the distribution of the weight and unweights group using the patients' characteristics.

**Table 1:** Baseline characteristics include method of morphine administration and category of analgesic prescription

	Analgesics prescribed as-needed (n = 963)						Analgesics prescribed preemptively (*n* = 568)					
Characteristics	IV morphine (*n* = 858)		Other analgesics (*n* = 105)		Absolute STD		IV morphine (*n* = 411)		Other analgesics (*n* = 157)		Absolute STD	
	*N*	%	*N*	%	Before	After	*N*	%	*N*	%	Before	After
Age (mean ± SD)	69.50	19.10	73.28	12.97			76.38	14.79	77.25	12.87		
Age > 70 years	608	70.86	74	70.48	0.013	0.075	334	81.27	126	80.25	0.003	0.001
Female	566	65.97	90	85.71	0.484	0,005	239	58.15	95	60.51	0.035	0.006
BMI (mean ± SD)	21.54	4.98	22.04	3.37	0.133	0.033	21.12	3.90	21.70	4.04	0.144	0.011
Body weight (mean ± SD)	53.49	12.49	52.42	09.47			52.30	12.10	53.28	10.62		
Height (mean ± SD)	157.40	9.08	154.91	7.18			157.35	8.35	156.84	8.51		
Femoral neck or subtrochanteric	387	45.10	52	49.52	0.051	0.012	169	41.12	61	38.85	0.041	0.002
Total or partial hip replacement	228	26.57	38	36.19	0.189	0.020	112	27.23	35	22.29	0.124	0.000
Operation time (median (IQR)	75	59,100	70	60,90	0.051	0,047	70	55, 90	65	55, 85	0.066	0,056
Spinal block	560	65.27	78	74.29	0.222	0.029	271	65.94	117	74.52	0.248	0.011

*IV* Intravenous, *SD* Standard Deviation, *BMI* Body Mass Index, *IQR* Interquartile Range, *Absolute STD* Absolute Standardized Difference

### Morphine dose and cost in the IV morphine group

The mean daily dose of IV morphine was 12 mg (IQR 9-18) in the prescribed as needed group and 9 mg (IQR 4-15) in the preemptive group. The mean dose of IV morphine in the first 24 h postoperatively was 12 mg (IQR6-15) in the prescribed as needed group and 7 mg (IQR 3-12) in the preemptive group. In comparison, the mean postoperative dose of IV morphine before rehabilitation was 12 mg (IQR 8-18) in the prescribed as needed group and 9 (IQR 4-15) in the preemptive group. The mean single dose of IV morphine 24 h postoperatively was 0.20 mg/kg (IQR 0.12–0.30) in the prescribed as-needed for pain control group. The overall mean cost of IV morphine per patient was 7.2 baht (IQR 5.4-10.8) (Additional file 2: Table S1).

### Hospital costs

The hospital costs of inpatient hip fracture treatment for each service unit are recorded in Tables 2 and 3. In the prescribed as-needed for the pain control group, the average total cost was statistically significantly different between the two groups: 75,898 baht (IQR 56,272–104,889) in the IV morphine group and 72,463 baht (range 60,828–100,941) in the other analgesics group (p = 0.036). The groups also had significant differences in diagnostic and radiological treatment and in-home medical fees. In the other analgesics group, the mean home medication cost was 533 baht (IQR 228,1,224), while in the IV morphine group, the mean was 314 baht (range 141–706) (*p* = 0.044). Mean diagnostic and radiological treatment was 1500 baht (IQR 1100–2200) and 1760 baht (IQR 1320–3150) (*p* = 0.026) in the other analgesics group and the IV morphine group, respectively. In the preemptive group, the average cost of room and food cost was statistically significantly different between the two groups: 10,000 baht (IQR 6500–14,380) in the other analgesics group and 7800 baht (IQR 4800–14,200) in the IV morphine group. Other costs showed no significant difference between the groups.

**Table 2:** Primary endpoint costs in patients for whom intravenous morphine was prescribed as-needed for pain control

Hospital cost (THB)	IV morphine (n− 929)		Other analgesics (n = 108)		Weighted median regression		
	Median	IQR	Median	IQR	Adjusted Effect	95% CI	*p*− value
Total cost	75,893	56,272, 104,889	72,463	60,828,100,941	5617	369.45 to 10,863.55	0.036
Technology and pathology	3420	2090,5550	3,588	2388,5040	65	− 344.81 to 474.81	0.756
Diagnostic and radiological treatment	1760	1320,3150	1,500	1100,2200	360	42.18 to 677.82	0.026
Procedure and anesthetic fee	10,990	3535,16,610	11,500	9900,16,310	275	− 1135.82 to 1685.82	0.700
Physical therapy and rehabilitation	17,550	11,700,36,400	15,600	7500, 18,000	1,300	− 14,654,26 to 17,254.26	0.860
Nursing service	6595	4490, 11,600	5,470	4090, 7510	790	− 138.35 to 1718.35	0.096
Non directly related medica treatment fee	1502	120,3977	9,950	2351,12,000	− 1,721	− 12,276.51 to 8834.514	0.745
Blood component	1,830	950, 2850	1.830	950,2780	0	− 697.50 to 697.60	1.000
Home medication fee	314	141,705	533	228,1224	− 274	− 540,76 to − 7.24	0.044
Parenteral drug	5537	3436,9679	5,538	3628,10,577	− 85	− 1196.33 to 1026,33	0.881
Room and food	6960	4400,12,600	8,800	5700,15,480	− 1,090	− 3125.58 to 965.68	0.300
Prosthesis and therapeutic	26,400	12,528, 34,400	26,400	12,734, 28,920	148	− 1684.87 to 1980,87	0.874
Medical equipment and supplies fees	1280	730, 2400	1,300	780, 2280	90	− 261,82 to 441.82	0.616
Nondrug medical supplies fee	2636	1663, 4299	2,200	1506, 3033	373	− 89.15 to 835.16	0.114

*IV* Intravenous, *IQR* Interquartile Range, *THB* Thai baht

**Table 3:** Primary endpoint costs in patients for whom intravenous morphine was prescribed preemptively

Hospital cost (THB)	IV morphine (n = 929)		Other analgesics (*n*− 108)		Weighted median regression		
	Median	IQR	Median	IQR	Adjusted Effect	95% CI	*p*− value
Total cost	82,269	62,908, 110,134	79,646	60,503,102,227	1,037	− 5842.38 to 7915.38	0.767
Technology and pathology	4,080	2490, 6500	3,605	2170, 5918	430	− 164.29 to 1024.29	0.156
Diagnostic and radiological treatment	1,890	1320, 3O80	1,840	1320,3160	140	− 147.01 to 427.01	0.338
Procedure and anesthetic fee	11,025	8500, 15,325	11,010	9000,15,190	275	− 944.15 to 1494.16	0.65S
Physical therapy and rehabilitation	13,000	7500,15,600	11,700	4500, 18,200	1,300	− 16,864.89 to 19,464.89	0.878
Nursing service	6,310	4630, 10,450	5,870	4310, 9040	700	− 229.62 to 1620.62	0.140
Non directly related medical treatment fee	1,495	60,13,500	3,401	300, 12,225	− 4,806	− 13,649.14 to 4037.14	0.277
Blood component	1,830	950, 2780	1,900	950,2930	− 70	− 558.45 to 418.45	0.778
Home medication fee	375	175, 961	46S	213, 1222	− 140	290.42 to 10,42	0.068
Parenteral drug	6,073	3396,12,304	6,552	3269,12,871	− 862	− 2386.84 to 662.84	0.267
Room and food	7,800	4800, 14,200	10,000	6500, 14,380	− 1640	− 3135.16 to − 144.84	0.032
Prosthesis and therapeutic	27,100	13,218, 34,400	26,786	12,528, 34,138	148	− 385.70 to 681.70	0.586
Medical equipment and supplies fees	1,750	1010, 3610	1,560	910,3070	200	− 113.22 to 513.22	0.210
Nondrug medical supplies fee	3,048	1925, 4496	2,870	1659, 3926	205	− 206.18 to 616.18	0.328

*IV* Intravenous, *IQR* Interquartile Range, *THB* Thai baht

### Amount and cost of other analgesic drugs

The amount and cost of each analgesic drug in the two groups are shown in Tables 4 and 5. There were no statistically significant differences between groups in the usage or cost of acetaminophen, combination drugs, non-selective NSAIDs, selective NSAIDs, or weak opioid drugs in the preemptive group. There was, however, a statistically significant difference in the weighted median regression cost of selective NSAIDs between the other analgesics group (mean 941 THB, IQR 477–1410) and the IV morphine group (mean 540 THB, IQR 280–1060) (*p* = 0.022), but not in the mean amount: (30.5 baht (IQR 18–52) in the other analgesics group and 20 baht (IQR10-40) in the IV morphine group (*p* = 0.19). The mean amount of acetaminophen consumed in the analgesics prescribed as needed group was statistically significantly higher in the other analgesics group than in the IV morphine group: 100 mg (IQR 64–120) and 82 mg (IQR 64–120) (*p* = 0.004), respectively. The difference in the mean cost of acetaminophen in the two groups was also statistically significantly different at 50 baht (IQR 32–70) and 46 baht (IQR 32–60), respectively.

**Table 4:** Drugs prescribed as needed for pain control

Hospital cost (THB)	IV morphine (*n* = 929)		Other analgesics (*n* = 108)		Weighted median regression		
	Median	IQR	Median	IQR	Adjusted Effect	95% CI	*p*− value
Acetaminophen							
Amount	82	64,120	100	64,120	− 18	− 30.14 to − 5.86	0.004
Cost	46	32,60	50	32,70	− 10	− 16.07 to − 3.93	0.001
Combined							
Amount	58	30,96	40	27,87	12	− 13.70 to 37.70	0.359
Cost	145	68,612	225	74,738	− 84	− 437,40 to 269.40	0.640
Nonselective NSAIDs							
Amount	34	12,56	24	12,60	8	− 9.43 to 25.43	0.367
Cost	39.5	18,72	48	22,76	− 16	− 43.50 to 11.50	0.253
Selective NSAIDs							
Amount	20	10,40	30.5	18,52	− 6	− 19.02 to 7.02	0.362
Cost	540	282,1060	941	477,1410	− 404	− 749.10 to − 58.89	0.022
Weak opioid							
Amount	4	2,41	10	2,22	− 6	− 15,17 to 3.17	0.199
Cost	86	20,296	130	41.5,261	− 60	− 166.18 to 46.18	0.267

*IV* Intravenous, *IQR* Interquartile Range, *THB* Thai baht

**Table 5:** Drugs prescribed preemptively

Hospital cost (THB)	IV morphine (*n* = 929)		Other analgesics (*n* = 108)		Weighted median regression		
	Median	IQR	Median	IQR	Adjusted Effect	95% CI	*p*− value
Acetaminophen							
Amount	80	62.120	80	60,120	0	− 8.25 to 8.25	1.000
Cost	42	36,60	40	32.62	2	− 2.12 to 6.12	0.341
Analgesics combined							
Amount	47	24,96	59	36.5,116	− 12	− 41.38 to 17.38	0.421
Cost	111	52,476	174	78.5,1049	− 78	− 468.67 to 312.67	0.694
Nonselective NSAIDs							
Amount	24	12,44	22	8,36	6	− 5.42 to 17.42	0.301
Cost	36	18,68	32	12,58	10	− 4.28 to 24.28	0.169
Selective NSAIDs							
Amount	18	6,30	24	12,50	− 6	− 24.98 to 12.98	0.529
Cost	456	222,990	700	318,1590	− 284	− 915.55 to 347.55	0.372
Weak opioid							
Amount	4	2,32.5	3	1.5,38	2	− 6.94 to 10.94	0.658
Cost	86	20,299	66	15,394	26	− 53.75 to 105.75	0.519

*IV* Intravenous, *IQR* Interquartile Range, *THB* Thai baht

### Postoperative pain score

Additional file 1: Fig. S1 shows the trends of postoperative pain scores in the other analgesics morphine group and the IV morphine group. The difference in pain scores between the two groups was not statistically significant at 72 h. For clarity, postoperative pain scores are classified into three categories: mild (NS < 5), moderate (NS 5–7), and severe (NS > 7).

### Adverse effects

Table 6 shows the incidence of adverse events in the other analgesics and the IV morphine groups in the prescribed as needed group. The mean number of patients who experienced gastrointestinal side effects was statistically significantly lower in the other analgesics group than in the IV morphine group (p < 0.01) with 206 (24%) and 11 (10.5%) patients, respectively. There was no significant difference between the groups for any other adverse events while Table 7, which represents the preemptive group, shows statistically significantly fewer events in the other analgesics group than in the IV morphine group (*p* < 0.01): 91 (22.14%) and 14 (8.92%) in the IV morphine group and other analgesics group, respectively.

**Table 6:** Side effects by method of morphine administration in the as-needed group

	IV morphine	Other analgesics	Weighted risk difference regression		
			Adjusted Effect	95% CI	*p*− value
GI	206(24)	11 (10.S)	0.17	0.11 to 0.23	< 0.001
MI	2 (0.23)	0 (0)	0.003	0.001 to 0.006	0.157
Symptomatic DVT	0 (0)	0 (0)	NE	NE	NE
Read mission	5 (0.58)	0 (0)	0.005	0.00 to 0.01	0.045
Wound infection	2 (0.23)	0(0)	0.003	− 0.001 to 0.006	0.158
Deep infection	0 (0)	0 (0)	NE	NE	NE
CVA	1 (0.12)	0 (0)	0.001	− 0.001 to 0.003	0.317
UTI	44 (5.13)	6 (5.71)	0.002	− 0.039 to 0.044	0.907
RTF	10 (1.17)	1 (0.95)	0.003	− 0.016 to 0.022	0.747
Delirium	29 (3.3B)	3 (2.86)	0.009	− 0.020 to 0.038	0.540

*IV* Intravenous, *NE* Not estimable, *GI* Gastrointestinal, *MI* Myocardial infarction, *DVT* Deep vein thrombosis, *CVA* Cerebrovascular accident, *UTI* Urinary tract infection, *RTI* Respiratory tract infection

**Table 7:** Side effects by method of morphine administration in the as-needed group

	IV morphine	Other analgesics	Weighted risk difference regression		
			Adjusted Effect	95% CI	*p*− value
GI	206(24)	11 (10.S)	0.17	0.11 to 0.23	< 0.001
MI	2 (0.23)	0 (0)	0.003	0.001 to 0.006	0.157
Symptomatic DVT	0 (0)	0 (0)	NE	NE	NE
Read mission	5 (0.58)	0 (0)	0.005	0.00 to 0.01	0.045
Wound infection	2 (0.23)	0(0)	0.003	− 0.001 to 0.006	0.158
Deep infection	0 (0)	0 (0)	NE	NE	NE
CVA	1 (0.12)	0 (0)	0.001	− 0.001 to 0.003	0.317
UTI	44 (5.13)	6 (5.71)	0.002	− 0.039 to 0.044	0.907
RTF	10 (1.17)	1 (0.95)	0.003	− 0.016 to 0.022	0.747
Delirium	29 (3.3B)	3 (2.86)	0.009	− 0.020 to 0.038	0.540

*IV* Intravenous, *NE* Not estimable, *GI* Gastrointestinal, *MI* Myocardial infarction, *DVT* Deep vein thrombosis, *CVA* Cerebrovascular accident, *UTI* Urinary tract infection, *RTI* Respiratory tract infection

## Discussion

This study investigated differences between IV morphine consumption and other analgesics consumption patients in terms of hospital costs, analgesic drug expense, postoperative complications, adverse analgesic effects, and functional outcomes. The participants in each group were subdivided into an as needed-for-pain control group (pain score ≥ 3 points) and a preemptive analgesic use group.

Patients prescribed IV morphine as-needed for pain control exhibited significantly higher total hospital, diagnostic, radiological treatment, and home medical fee costs than the other analgesic group. Both preemptive analgesic sub-groups demonstrated statistically significant differences only in room and food costs. In contrast, total hospital costs were notably higher in the as-needed IV morphine group, with a mean cost of approximately 75,898 THB (US $2,277 vs. 72,463 bath (US$ 2,174), respectively. Notably, the cost of diagnostics, radiological treatment, room and food, and home medication fees were significantly higher in the as-needed IV morphine group. Globally, the pooled mean cost for the index hospitalization was US$10,075 (95% CI range $8,322-$11,838) [333,483 THB, (275,458–392,198 THB)] [24], with the mean cost of IV opioids for orthopedic surgery averaging around $25–$27 (828–894 THB) in most country [25]. Preoperative and intraoperative costs, including diagnostic, radiological and anesthetic, prosthesis and therapeutic fees are also not significantly different.

In this study, the cost of diagnostics, radiological treatment, room and food, and home medication fees were significantly higher in the IV morphine were prescribed as-needed for pain control group than the other analgesics. Factors that could potentially explain these results include higher demands on services related to pain and its side effects in the IV morphine group and the limitation of analgesic choices regarding the patients’ health coverage package. The cost of home medication was also higher in the other analgesics group because that group received more oral drugs for pain control. Costs for inpatient hip fracture treatment can be incurred in the areas of acute stay, operative treatment, laboratory investigation, and radiology. Acute stay costs, including nursing service, room, and food, are the main factors, accounting for about 84% of total inpatient costs [26]. However, there have been no studies of why the costs of nursing service, room, and food are higher for other analgesic hip fracture patients than for IV patients.

No difference was found between IV and other analgesics patients in the use of all analgesic drugs in the preemptive drug group or in the use of other drugs, including combination drugs, non-selective NSAIDS, and weak opioids for controlling pain. The difference in the consumption and cost of acetaminophen and selective NSAIDs identified in this study is a reflection of a limitation of the government health care coverage system in Thailand. Currently, the most widely used public health protection schemes available to all Thai citizens are the Universal Health-care Coverage Scheme (UCS) which is available to about 76% of the population followed by the Social Security Scheme (SSS) at 15%. In-patient services are reimbursed using a case-mixed system, Diagnostic Related Groups (DRG). These systems limit the amount of money paid to hospitals [27, 28].

Gastrointestinal, respiratory, and genitourinary-related complications are the most frequent adverse events associated with orthopedic procedures [23]. GI side effects is a statistically significant difference and were significantly more frequent in the IV morphine group, although that result may not reflect an actual cause-and-effect relationship. Both NSAIDs and opioids have been shown to involve a high risk of gastrointestinal side effects [29]. Use of opioids can result in sedation, apnea, nausea, vomiting, and constipation meditated in the brain or gut, while nonsteroidal anti-inflammatory drugs can elicit gastrointestinal ulcers and bleeding [17, 30]. The most common side effect in the other analgesics group was gastritis, while in the IV morphine group it was nausea and vomiting. However, weak opioids within other analgesic groups exhibit similar side effects to strong opioids, such as constipation, nausea, vomiting, sedation, dizziness, respiratory depression, and seizures. The incidence, severity, and specific manifestations of these side effects can be influenced by the particular opioid used. In this study, the IV morphine group had a higher incidence of GI side effects, presumably because their medication contained both NSAIDs and opioids.

This study found only a limited association between potential risk factors and immediate postoperative pain after hip fracture surgery, unlike a previous study which reported lower education level (< 8 yrs of school) and the presence of psychological depression as being significantly associated with severe postoperative pain [31]. A detailed analysis of these risk factors could not be included in this study because of the limitations of documentation available in the hospital database. Investigation of predictive factors for postoperative pain in hip fracture patients will require further study.

## Conclusion

Treatment of hip fractures with either intravenous morphine or other analgesics appears to be associated with increased use of NSAIDs and/or acetaminophen as well as the incidence of GI side effects.

The decision to treat with either intravenous or other analgesics may also significantly affect medical expenses and hospitalisation outcomes. Additional studies of these relationships are needed, especially in different geographic and political settings.

### Supplementary Information

**Additional file 1.** Average pain score over time.

**Additional file 2.** Table of morphine comsumption.

## Data Availability

The analyzed data sets generated during the current study are available from the corresponding author on reasonable request.

## Abbreviations

Absolute STD: Absolute standardized difference
BMI: Body mass index
DRG: Diagnostic related groups
GI: Gastrointestinal
IQR: Interquartile range
IV: Intravenous
NE: Not estimable
NSAIDs: Non-steroidal anti-inflammatory
NS: Numeric scale
PT: Physical therapy
SD: Standard deviation
SSS: Social security scheme
THB: Thai Baht
UCS: Universal coverage scheme
