# Correction: Outcomes of hip fracture treatment with intravenous morphine and with other analgesics: postoperative analgesic medical expense, severity of pain and hospitalisation—a retrospective study

**DOI:** 10.1186/s13018-023-04460-7

**Published:** 2023-12-20

**Authors:** Rapeepat Srichan, Phichayut Phinyo, Krittai Tanasombatkul, Puwapong Nimkingratana

**Affiliations:** 1 Department of Orthopaedics, Faculty of Medicine, Chiang Mai University, Chiang Mai, Thailand; 2 Center for Clinical Epidemiology and Clinical Statistics, Faculty of Medicine, Chiang Mai University, Chiang Mai, Thailand; 3 Department of Family Medicine, Faculty of Medicine, Chiang Mai University, Chiang Mai, Thailand

**Correction: Journal of Orthopaedic Surgery and Research (2023) 18:925** 10.1186/s13018-023-04328-w

Following publication of the original article [1], the authors identified an error in the author name of Phichayut Phinyo.

The incorrect author name is: Pichayut Phinyo

The correct author name is: Phichayut Phinyo

The author group has been updated above and the original article [1] has been corrected.

## References

[1] Srichan R, Phinyo P, Tanasombatkul K, Nimkingratana P (2023). Outcomes of hip fracture treatment with intravenous morphine and with other analgesics: postoperative analgesic medical expense, severity of pain and hospitalisation—a retrospective study. J Orthop Surg Res.

