# Supplementary figures and images for: Outcomes of hip fracture treatment with intravenous morphine and with other analgesics: postoperative analgesic medical expense, severity of pain and hospitalisation—a retrospective study
Source: J Orthop Surg Res. 2023 Dec 6;18:925. doi: 10.1186/s13018-023-04328-w (PMC10698994; doi:10.1186/s13018-023-04328-w)

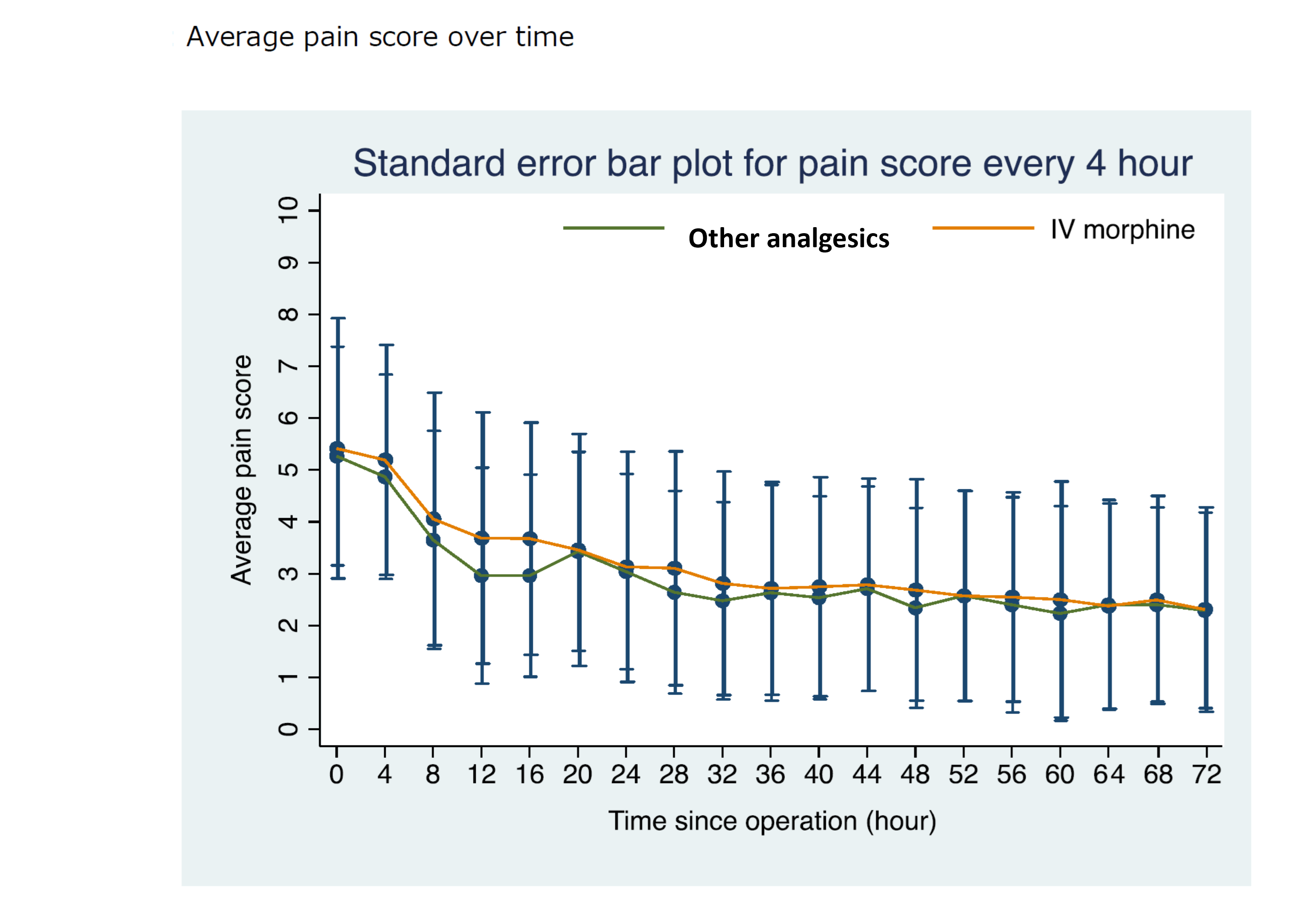

Supplement: Supplementary file 1 — Additional file 1. Average pain score over time. [file 13018_2023_4328_MOESM1_ESM.tiff]

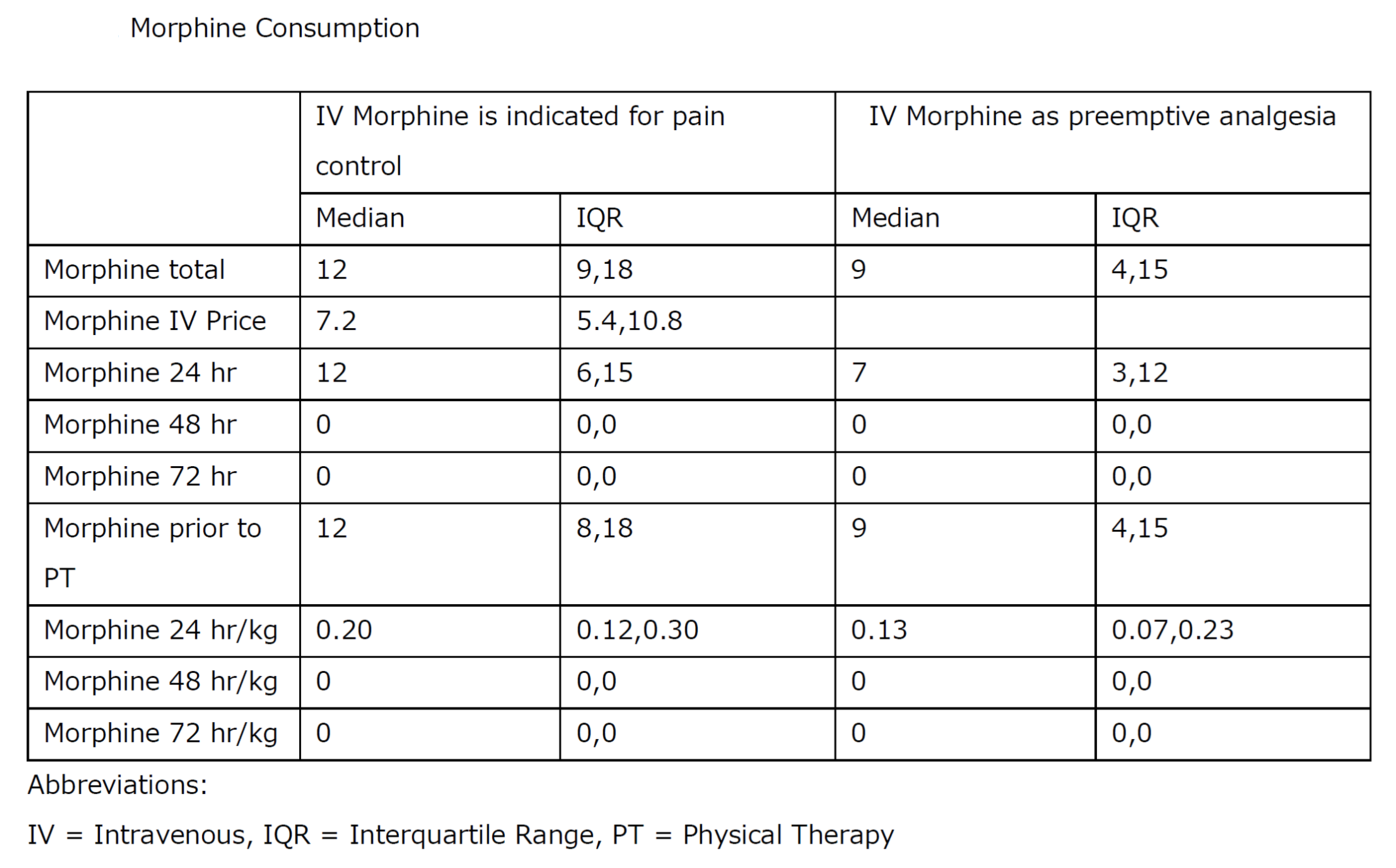

Supplement: Supplementary file 2 — Additional file 2. Table of morphine comsumption. [file 13018_2023_4328_MOESM2_ESM.tiff]
